# Sepsis induces long-lasting impairments in CD4^+^ T-cell responses despite rapid numerical recovery of T-lymphocyte populations

**DOI:** 10.1371/journal.pone.0211716

**Published:** 2019-02-07

**Authors:** Christoph Ammer-Herrmenau, Upasana Kulkarni, Nico Andreas, Martin Ungelenk, Sarina Ravens, Christian Hübner, Angela Kather, Ingo Kurth, Michael Bauer, Thomas Kamradt

**Affiliations:** 1 Institute of Immunology, Jena University Hospital, Jena, Germany, United States of America; 2 Institute of Human Genetics, Jena University Hospital, Jena, Germany; 3 Institute of Immunology, Hannover Medical School, Hannover, Germany; 4 Institute of Human Genetics, Medical Faculty, RWTH Aachen University, Aachen, Germany; 5 Department of Anesthesiology and Intensive Care Medicine, Jena University Hospital, Jena, Germany; 6 Center for Sepsis Control & Care, Jena University Hospital, Jena, Germany; Universita Cattolica del Sacro Cuore, ITALY

## Abstract

Massive apoptosis of lymphocytes is a hallmark of sepsis. The resulting immunosuppression is associated with secondary infections, which are often lethal. Moreover, sepsis-survivors are burdened with increased morbidity and mortality for several years after the sepsis episode. The duration and clinical consequences of sepsis induced-immunosuppression are currently unknown. We have used the mouse model of peritoneal contamination and infection (PCI) to investigate the quantitative and qualitative recovery of T lymphocytes for 3.5 months after sepsis with or without IL-7 treatment. Thymic output and the numbers of naive and effector/memory CD4^+^ and CD8^+^ lymphocytes quickly recovered after sepsis. IL-7 treatment resulted in an accelerated recovery of CD8^+^ lymphocytes. Next generation sequencing revealed no significant narrowing of the T cell receptor repertoire 3.5 months after sepsis. In contrast, detailed functional analyses of T helper (Th)-cell responses towards a fungal antigen revealed a significant loss of Th cells. Whereas cytokine production was not impaired at the single cell level, the absolute number of Th cells specific for the fungal antigen was reduced. Our data indicate a clinically relevant loss of pathogen-specific T cell clones after sepsis. Given the small number of naive T lymphocytes specific for a given antigen, this decrement of T cell clones remains undetected even by sensitive methods such as deep sequencing. Taken together, our data are compatible with long lasting impairments in CD4^+^ T-cell responses after sepsis despite rapid recovery of T lymphocyte populations.

## Introduction

Sepsis is defined as life-threatening organ dysfunction caused by a dysregulated host response to infection [1]. Epidemiological studies suggest that more than 30 million cases of sepsis occur annually world-wide [2]. In the U.S., sepsis is the most expensive disease treated in hospitals with estimated hospital costs of > 20 billion US $ annually [3]. Mortality rates have been declining in high-income countries due to improved treatment and range from 20–50% depending on disease severity and other factors [4,5]. Immunologically, sepsis is characterised by concurrent proinflammatory and immunosuppressive alterations [6–10]. A prominent feature contributing to immunosuppression in sepsis is an early massive loss of lymphocytes due to apoptosis [8–12], which is recapitulated in mouse models of sepsis [8–10,13]. Profound or persistent lymphopenia in sepsis patients is associated with increased mortality [14,15]. Another important mechanism of sepsis-induced immune-suppression is the expansion of immunosuppressive cell populations including regulatory T lymphocytes, IL-10-producing B lymphocytes and myeloid-derived suppressor cells (MDSC) [8–10,16]. We have recently shown that numbers of IL-10 producing B lymphocytes and MDSC remain increased for months after sepsis [16]. Sepsis-induced immune-suppression renders patients susceptible to secondary opportunistic infections [17,18] and reactivation of latent viral infections [19], both of which contribute to late sepsis mortality [4,5,20]. It is currently unclear how long the sepsis-induced immunosuppression lasts and if an immunological *restitutio ad integrum* is reached in sepsis survivors. Most clinical and experimental studies to date have focussed on the immunopathology of acute sepsis. Clinical and epidemiological data indicate a massively increased morbidity and mortality of sepsis survivors for years after discharge from the hospital [4,21–23] and it is currently unknown how much persistent immunological alterations contribute to this disease burden. Boosting the immune response in sepsis patients is a promising approach to improve survival [4,9,10]. One candidate approach is the cytokine Interleukin (IL)-7, which is important for T-cell survival [24]. Early IL-7 treatment has been shown to improve survival in murine sepsis models [25,26] and to restore normal lymphocyte counts and functions in septic patients [27,28]. On the other hand, late IL-7 treatment prolongs the sepsis induced expansion of immunosuppressive IL-10 producing B-lymphocytes and MDSC after sepsis [16].

We therefore studied the long-term recovery of different T cell subsets after sepsis with or without IL-7 treatment in the model of peritoneal contamination and infection (PCI) [16,29]. We analyzed the recovery of naive and effector/memory CD4^+^ and CD8^+^ T cell subsets and analyzed thymic output and T-cell receptor (TCR)-repertoire diversity 1 week, 1 month and 3.5 months after sepsis induction, representing the post-acute, late and very late time points, respectively. At 30 days after sepsis we also immunised mice with a fungal antigen and analyzed the T-cell response quantitatively and qualitatively.

## Materials and methods

### Mice

C57BL/6 mice and B6.Rag2-GFP mice [30] were bred and maintained at the animal facility of the University Hospital Jena. At the end of the experiments mice were killed by cervical dislocation under CO_2_ anaesthesia. All animal experiments were approved by the appropriate governmental authority (Thüringer Landesamt für Lebensmittelsicherheit und Verbraucherschutz; Bad Langensalza, Germany; registration number 02–007/14) and conducted in accordance with institutional and state guidelines: Efforts to alleviate suffering included regular inspection of the mice and the definition of humane enpoints according to institutional and state guidelines.

### Sepsis–induction and treatment

Sepsis was induced in mice by peritoneal contamination and infection (PCI) as described previously [16,29]. Briefly, human stool samples from three healthy non-vegetarian donors were collected, prepared and stored at -80°C Composition and number of CFUs for the different pertinent strains after storage were determined by microbiologic analysis as described in detail in [29]. Animals were randomly allocated to the sepsis or sham group. Sepsis was induced by intraperitoneal (i.p.) injection of 1.75 ml/kg body weight stool suspension, diluted (1:4) in saline. Control mice received the same volume of saline i.p. (sham group). Beginning at 7 hours after sepsis induction, the infected mice were treated with meropenem (12 mg/kg, subcutaneous (s.c.) twice daily for 3 days. They were evaluated for conjunctivitis, diarrhea, weakness and lack of movement. As expected [29], approximately 40–50% of the mice died within the acute phase (d1-5 post sepsis-induction) [16]. The following endpoints were defined and approved by the relevant authorities (Thüringer Landesamt für Lebensmittelsicherheit und Verbraucherschutz; Bad Langensalza, Germany registration number 02–007/14): Lack of movement (even after touching), ascites, minimal signs of respiratory distress, severe conjunctivitis, severe diarrhea, cutaneous abscess (injection abscess). Once a mouse reached one of the defined endpoints it was immediately euthanized. No animals died before meeting criteria for euthanasia. Animal health and behavior was monitored at day of sepsis induction every 4 hours, the following 2 days every 6 hours and at all other days at least once a day. All people handling mice have had special training, either as professional animal care takers or–upon successful completion of a mandatory training course–a certification approved by the appropriate governmental authority (Thüringer Landesamt für Lebensmittelsicherheit und Verbraucherschutz; Bad Langensalza, Germany) registration number 02–007/14). In total around 580 animals were used, 186 were euthanized, no found dead. Cause of death was always consequences of sepsis.

On d5 the surviving mice were randomly allocated to receive IL-7 or no further treatment. Recombinant human IL-7 (R&D Systems), which is known to bind and signal via the mouse IL-7 receptor [31], was subcutaneously injected (2.5 μg/mouse/day) from d5 post sepsis induction for the following 6 days. In order to stabilize the cytokine, IL-7 was precipitated with a ten-fold higher concentration of an anti-human IL-7 antibody (clone M25; BioXCell) [32].

### Immunization with Aspergillus antigen

The *Aspergillus* antigen gliotoxin sulfhydryl oxidase (GliT) was produced as described [33] and kindly provided by D.H. Scharf and O. Kniemeyer (Hans Knöll Institute, Jena). GliT is an enzyme involved in the biosynthesis of the mycotoxin gliotoxin by the filamentous fungus *Aspergillus fumigatus*. The protein was dissolved in 50 mM potassium phosphate buffer (pH 7.0) / 50% glycerol. Mice were immunized subcutaneously with GliT in complete Freund’s adjuvant (CFA Sigma) at d30 after sepsis induction. One week later the mice were sacrificed, the spleens were harvested and the antigen-specific T cells analyzed.

### Cell culture and flow cytometry

Spleens were isolated and single cell suspensions were obtained by passing through strainers (BD Falcon). Erythrocytes were lysed using ammonium chloride solution. Cells were counted in a Neubauer chamber. Absolute numbers of cell populations were calculated by multiplying the total spleen cell count with the frequency of the respective population as determined by flow cytometry.

For flow-cytometric analyses, cells were stained with live/dead fixable aqua dead cell stain kit (Invitrogen) according to the manufacturer’s protocol. Cells were incubated with anti-CD16/CD32 (clone 2.4G2, in house production) and fluorophore-conjugated antibodies directed against surface antigens, CD3 (clone 145-2C11, in house production), CD4 (clone GK1.5; eBioscience); CD8 (clone YTS169.4, in house production); CD62L (clone MEL14, in house production); CD154 (clone MR, Miltenyi Biotec) CD44 (clone IM7, BioLegend).

GLiT-specific T cells were analyzed following stimulation of 1 x 10^7^/ mL lymph node or spleen cells with 3μg/ml anti-mouse CD28 (clone 37.51) and 200μg/ml aspergillus antigen GLiT for 6 hours in a humidified incubator at 37°C and 5%CO_2_. After 2 hours of incubation 1μl Brefeldin A (5μg/ml AppliChem A2138.0025) was added to each sample for the final 4 hours. GLiT-specific T cells were fixed in buffer containing 2% paraformaldehyde, permeabilized with buffer containing 0.5% saponin and stained with fluorophore-conjugated monoclonal antibody against tumor necrosis factor (TNF)-α (MP6-XT22l; eBioscience), IL-17 (TC11-18H10.1; BioLegend), IFNγ (clone XMG1.2, Bio X Cell), IL-10 (clone JES5-16E3, BioLegend) and granulocyte-macrophage colony stimulating factor (GM-CSF) (MP1-22E9, eBioscience).

Data from the samples were collected with a LSR II (BD Bioschiences) and analyzed using FlowJo software (TreeStar Inc.). Cytokine-expressing cells were identified by hierarchical gating for living, living CD4^+^, and living CD4^+^CD154^+^ cells. Cytokine patterns among cells were analyzed by boolean gating on either TNFα -positive cells or NOT-TNFα-positive cells and subsequent analysis of quadrant gating on IFNγ ^+^IL-17^-^, IFNγ ^+^IL-17^+^, IFNγ ^-^IL-17^+^, or IFNγ ^-^IL-17^-^ cells.

### T-cell receptor (TCR) diversity analysis

Mice were sacrificed and spleens were harvested. Total RNA was extracted using Trizol Reagent (Ambiom) from 10^7^ unsorted spleen cells of every septic and sham mouse. mRNA was purified with Direct-zol RNA MiniPrep (ZymoResearch). Complementary (c)DNA synthesis was performed with M-MLV reverse Transcriptase (Affymetrix), RNasin Plus (Promega) and reverse transcriptase Primer (C1 segment of β-chain, Biotez) according to the manufacturers’ recommendations. To generate templates for the High-throughput (HT) sequencing, the cDNA was amplified with primers spanning the whole complementarity-determining region (CDR)3 starting from 5´ Vβ 1,2,3,5,9,12–2,13–1,13–2,13–3,14,16,17,19,20,26 and from 3´ with the C1 segment of the β-chain (all primers were purchased from Biotez). Multiplex PCR was performed using a Multiplex PCR Kit (Qiagen) and finally purified with agarose gel electrophoresis and a Gel DNA-Recovery Kit (Zymoresearch). Subsequently, the Nextera XT library preparation kit (Illumina) was used to generate a sequence ready library according to the manufacturer’s protocol. The library was run on a MiSeq Sequencer (Illumina) with a 300 cycle MiSeq reagent kit v2 (Illumina). Obtained sequences were annotated by IMMUNOGENETICS (IMGT). All downstream data analysis was done using in-house bash and R scripts (version 3.2.2), which are available upon request. First, only productive amino acid sequences were taken for further analysis. Clones were identified based on identical CDR3 sequences. In addition, all results were represented as percentages of productive reads to normalize between samples. For further calculations, samples were normalized to 50000 reads and analyzed using the library vegan (cran.r-project). Trimmed fastq-data and the products of R-script were uploaded to ArrayExpress (E-MTAB-7483).

### Statistic evaluation and graphs

All data were statistically evaluated using SPSS. Comparisons involving multiple groups were analyzed in a two-stage procedure by one-way ANOVA. If the ANOVA indicated a significant difference between the groups (*P* < 0.05), all groups were further compared pairwise by Tukey's multiple comparison test. * *P* < 0.05, ** *P* < 0.01, *** *P* < 0.001. Data are expressed as mean ± SEM as indicated in the figure legends. All graphs were created by using SigmaPlot.

## Results

### Lymphopenia and recovery of T-lymphocyte numbers after sepsis

T lymphocytes undergo apoptosis in both septic animals [13] or patients [11,34,35]. To determine the quality and kinetics of T-lymphocyte recovery after experimental sepsis we examined T lymphocytes in the secondary lymphatic organs of mice for 3.5 months after sepsis induction in the PCI model. In addition, mice were randomly allocated to the IL-7 treatment group, which was treated subcutaneously with 2.5 μg recombinant human IL-7 daily from d5–9 post sepsis induction, or the control group, which received no further treatment.

As described earlier [16], the mortality within the first five days after sepsis induction was >40%. Thereafter mortality was approximately 10% within the following 30 days and similar in IL-7 treated or non-treated mice throughout observation period of 3.5 months.

**Fig 1:**
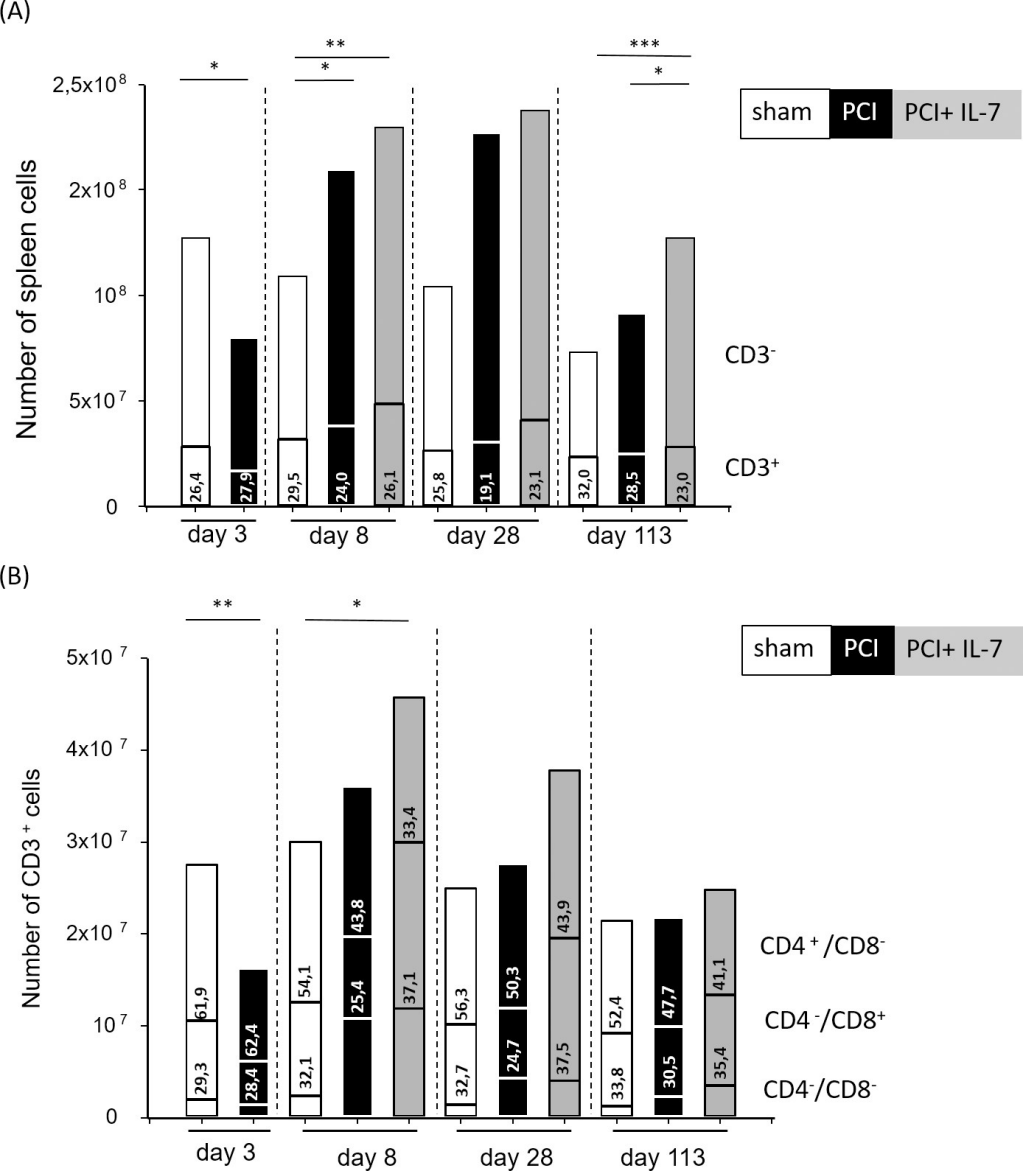
The loss of T lymphocytes in acute sepsis is followed by a rapid and sustained increase in T-lymphocyte numbers. Mice were injected with PBS i.p. (sham) or subjected to sepsis induction (PCI) or sepsis-induction plus IL-7 treatment (PCI + IL-7). At the indicated time points between d3 and d113 after PCI at least 6 mice per group were sacrificed and splenic cell numbers determined. **(A)** absolute cell numbers and frequencies of CD3^+^ T lymphocytes in the spleen; **(B)** numbers and frequencies of CD4^+^ and CD8^+^ subpopulations of CD3^+^ T lymphocytes in the spleen.

In sham-treated mice the total number of cells in the spleen did not change during the first four weeks of observation. At d113 the number of cells was significantly lower (p <0.005), compatible with the known effects of aging in mice [36–38] (**Fig 1A**). In septic mice there was a dramatic decrease of spleen cells at day d3 after sepsis induction. Thereafter, cell numbers quickly recovered and were even significantly higher than in sham mice at d8 after sepsis induction. IL-7 treatment from d5-9 resulted in significantly higher spleen cell numbers as compared with non-treated septic mice only at the very late time point (d113) after sepsis induction (**Fig 1A**).

The frequency (**Fig 1A**) and number (**Fig 1B**) of CD3^+^ T lymphocytes in the spleen was nearly constant in the sham mice during the observation period. In septic mice, a reduction of CD3^+^ T lymphocytes occurred at d3 after sepsis induction as expected [13]. Numbers and frequencies of T lymphocytes quickly recovered thereafter and were similar to those in sham-treated mice from d8 through d113 (**Fig 1B**). In contrast, the initial loss of lymphocytes was more than compensated in IL-7 treated septic mice, which harboured significantly more CD3^+^ cells in their spleen compared with sham mice d8 after sepsis induction (**Fig 1B**). This increase was still detectable, however no longer significant, at d28 and at d113, when the numbers and frequencies of T lymphocytes were similar in all three groups (**Fig 1B**). The percentage and number of CD3^+^CD4^-^CD8^-^T lymphocytes was strongly increased in sepsis-surviving mice at day 8 after PCI with or without IL-7 treatment (see also [16]). Moreover, there was a massive increase in CD3^+^CD8^+^ T cells after sepsis in the IL-7 treated mice (**Fig 1B**). The numbers and frequencies of CD4^+^ T cells largely reflected the number and frequencies of all T lymphocytes: a massive drop at d3 after sepsis induction was followed by a rapid reconstitution while no significant difference in CD4^+^ T cell numbers between the three groups of mice was recognised.

### Recovery of naive and effector T cells after sepsis

**Fig 2:**
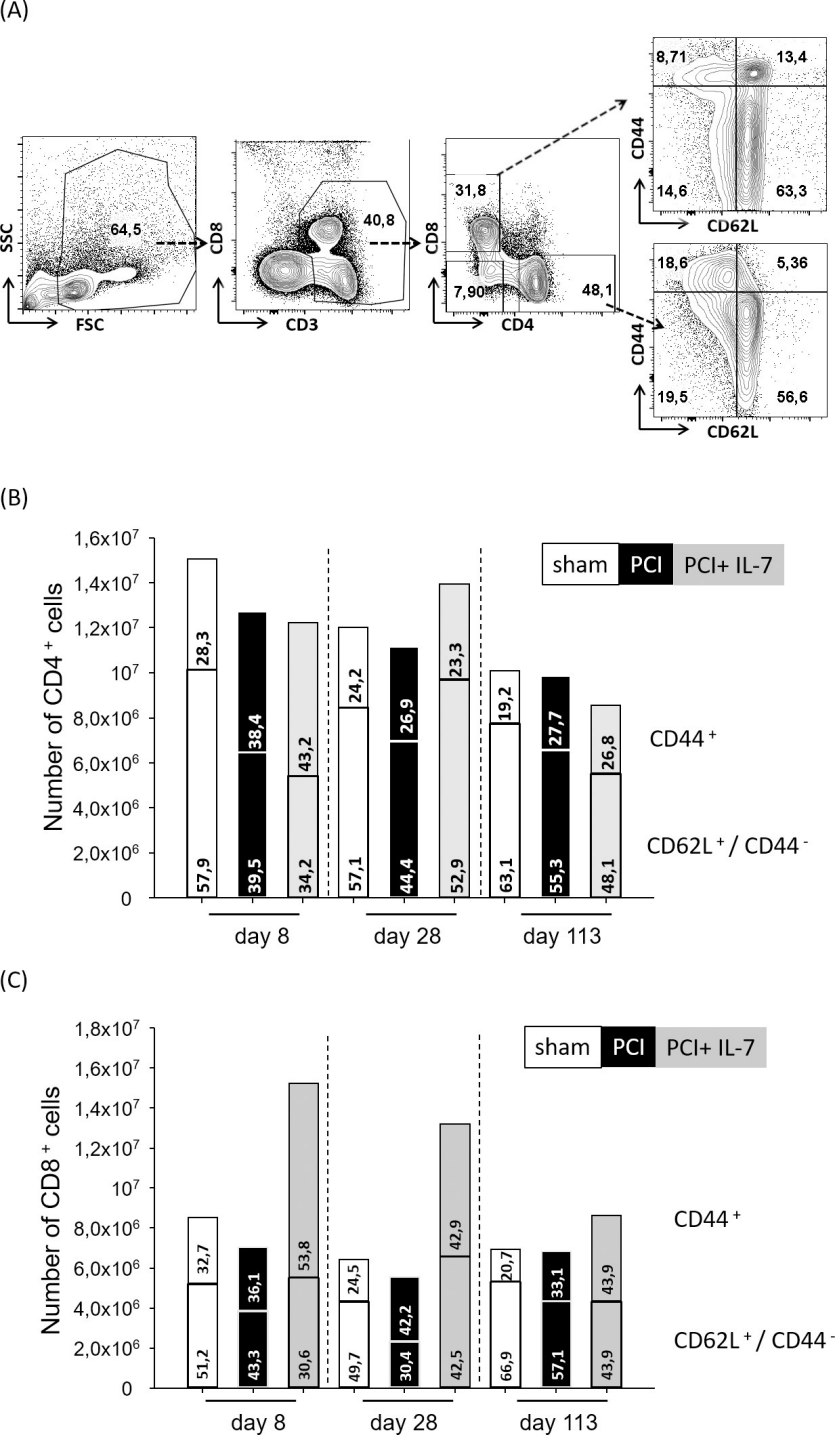
Recovery of naive and effector/memory T lymphocytes after sepsis. Mice were injected with PBS i.p. (Sham) or subjected to sepsis induction (PCI) or sepsis-induction plus IL-7 treatment (PCI + IL-7). At the indicated time points between d8 and d113 after PCI at least 6 mice per group were sacrificed and spleen cells analyzed by flow cytometry. **(A)** CD3^+^CD4^+^ T cells and CD3^+^CD8^+^ T cells were analyzed for their expression of CD62L and CD44 and categorized as naive cells (CD62L^hi^CD44^-^) or effector/memory cells (CD44^+^CD62L^-^ or CD44^+^CD62L^+^). **(B-C)** Longitudinal analysis of numbers and frequencies of **(B)** naive and effector/memory CD4^+^ T cells or **(C)** naive and effector/memory CD8^+^ cytotoxic T cells.

The survival and homeostasis of naive and effector T cells are controlled by different mechanisms [39,40]. We examined the long-term consequences of sepsis on CD4^+^ and CD8^+^ naive (CD62L^hi^ CD44^-^) and effector/memory (CD44^+^CD62L^lo or +^) T lymphocytes (**Fig 2A**). At d8, both the number and the percentage of naive CD4^+^ T cells were significantly lower in septic mice than in sham-mice, regardless of whether the septic mice were IL-7 treated or not (**Fig 2B**). In septic mice, numbers and frequencies of naive CD4^+^ T cells quickly recovered thereafter and were not significantly different from those in control mice. In IL-7-treated septic mice the recovery of naive CD4^+^ T cells occurred faster than in the septic mice that had not received IL-7. 113 days after sepsis induction the numbers and frequency of naive CD4^+^ T cells were similar in all three groups of mice. Immediately after sepsis, at day 8 after PCI, the number of CD8^+^ T cells was significantly higher in the IL-7 treated septic mice than in the two other groups (**Fig 2C**). This was almost exclusively due to a significantly higher number and frequency of CD8^+^ effector memory cells in the IL-7 treated mice. At d28 after PCI the number of CD8^+^ T cells was still significantly higher in the IL-7 treated group of septic mice, again mostly due to an increase in effector/memory cells. At d113 post PCI the frequency and numbers of CD8^+^ effector/memory cells were significantly higher in the IL-7 treated group compared with the sham-treated mice. Taken together, the numbers and frequencies of CD4^+^ and CD8^+^ naive and effector/memory cells quickly recovered and there were no significant differences among the three groups of mice except for a higher number and frequency of CD8^+^ effector/memory cells in IL-7 treated septic mice.

### Sepsis causes a transient reduction in thymic output

**Fig 3:**
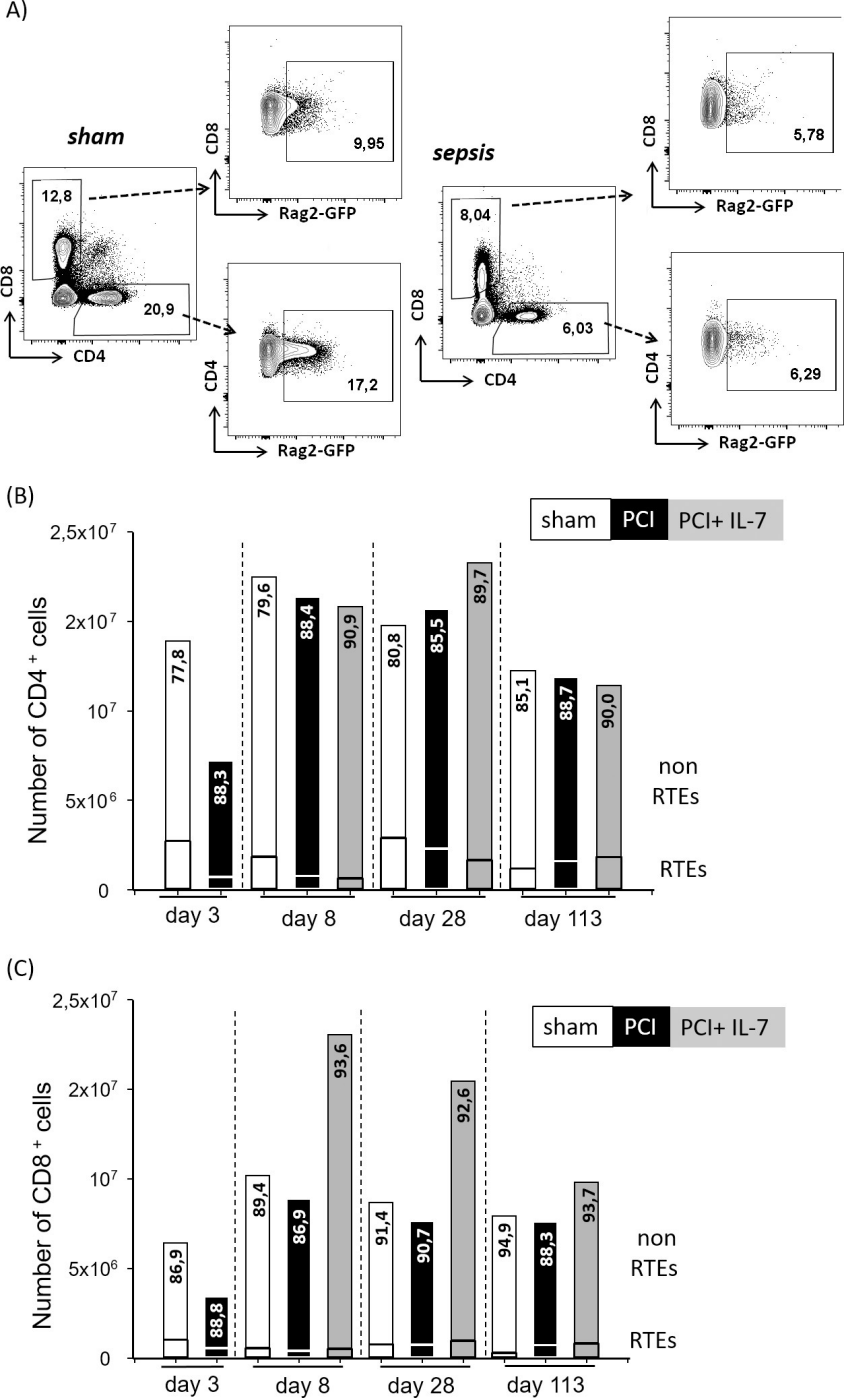
Repopulation of the peripheral T cell pool by recent thymic emigrants (RTEs) after sepsis. B6.Rag2-GFP mice were injected with PBS i.p. (sham) or subjected to sepsis induction (PCI) or sepsis induction plus IL-7 treatment (PCI + IL-7). At the indicated time points between d3 and d113 after PCI, at least 6 mice per group were sacrificed and spleen cells analyzed by flow cytometry. **(A)** RTEs within the CD4^+^ or CD8^+^ lymphocyte population were identified by their expression of green fluorescent protein. **(B, C)** Longitudinal analysis of numbers and frequencies of RTEs among **(B)** T cells or RTEs among **(C)** cytotoxic T cells.

Our data thus far have shown that the overall number of T lymphocytes as well as the CD4^+^ and CD8^+^ subpopulations including the naive T cells have largely returned to normal values 3.5 months after sepsis. The recovery of T cell populations could be due to increased thymic output after sepsis or to homeostatic proliferation of those T cell clones which survived during sepsis. We used RAG2p-GFP transgenic mice [30] to examine thymic output after sepsis (**Fig 3A**). In these mice the RAG2 promoter drives green fluorescent protein (GFP) expression and the GFP signal remains detectable for some time even after the RAG gene is no longer expressed [41]. In accordance with the literature [42] approximately 20% of the CD4^+^ T cells in the spleen of sham mice were recent thymic emigrants (RTEs); at d113 this frequency was slightly lower at 15% (**Fig 3B**). The percentage of CD4^+^ RTEs was reduced to approximately 11% in septic mice at d3 and d8 after PCI. Given that cell numbers were similar in septic and sham mice at d8, the reduced percentage of RTEs indicates homeostatic proliferation of surviving clones. The number of RTEs slowly increased thereafter and the difference between sham mice and septic mice was no longer significant at d28 and d113 after PCI. The treatment of septic mice with IL-7 did not have a significant impact on the number of CD4^+^ RTEs (**Fig 3B**). Regarding the frequency of CD8^+^ RTEs there was no significant difference between septic mice and controls at any time point (**Fig 3C**). IL-7 treatment did not influence the number of CD8^+^ RTEs (**Fig 3C**).

### Next generation sequencing (NGS) reveals no global decrease in T-cell receptor (TCR)-repertoire diversity in post-septic mice

**Fig 4:**
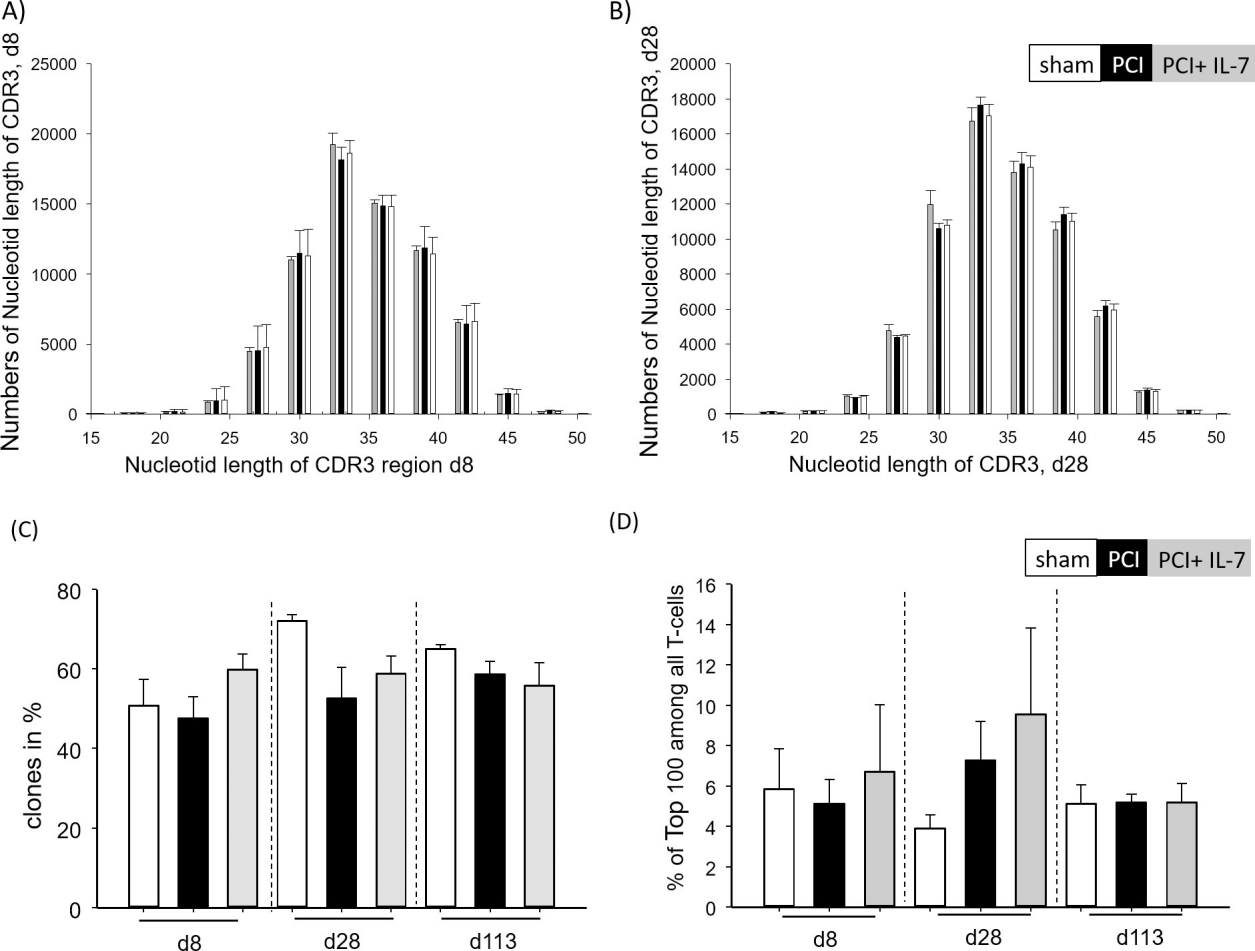
Analyses of TCR diversity after sepsis. Mice were injected with PBS i.p. (sham) or subjected to sepsis induction (PCI) or sepsis-induction plus IL-7 treatment (PCI + IL-7). At the indicated time points between d8 and d113 after PCI at least 4 mice per group were sacrificed, the spleens were harvested and TCRs were sequenced and analyzed as described in *Materials and Methods*. **(A)** CDR3 length (nucleotides) of T cells at day 8 after PCI. **(B)** CDR3 length (amino acids) of T cells at day 28 after PCI. **(C)** percentage of unique clones among all TCR transcripts analyzed. **(D)** Percentage of the 100 most frequently occurring clonotypes among all TCR transcripts analyzed.

We next asked whether the transient reduction in thymic output resulted in a less diverse TCR repertoire in mice that had survived sepsis. To obtain a more detailed picture of TCR diversity we used next generation sequencing for high-throughput analyses of the TCR-repertoires. First, we examined the clonal composition of CD3^+^ T lymphocytes by determining the distribution of CDR3 lengths (spectratyping). Gaussian distributions of CDR3 lengths were observed d8 (**Fig 4A**) and d28 (**Fig 4B**) after PCI. To assess T-cell receptor diversity we chose the percentage of unique clonotypes in 50.000 cDNAs generated from individual mice, which has been demonstrated to correlate strongly with other widely used approaches e.g. the Shannon-Wiener index or the Chao1 index [43]. At all analyzed time points the percentage of individual clonotypes in unsorted T cells varied between 50 and 70% and there was no significant difference between the three groups of mice (**Fig 4C**). To examine further if there was preferential clonal outgrowth after sepsis we analyzed the portion of the repertoire occupied by the 100 most frequently occurring TCR sequences at d8, d28 and d113 after PCI. Similar to earlier findings [43], the share of the 100 top-frequency clonotypes was 4–10% with no significant differences between the three groups or time-points (**Fig 4D**). Taken together, global analyses of TCR transcript diversity did not reveal significant differences between control mice and septic mice that had either received IL-7 treatment or not.

### Blunted T-cell responses against fungal antigen in mice that had survived sepsis

**Fig 5:**
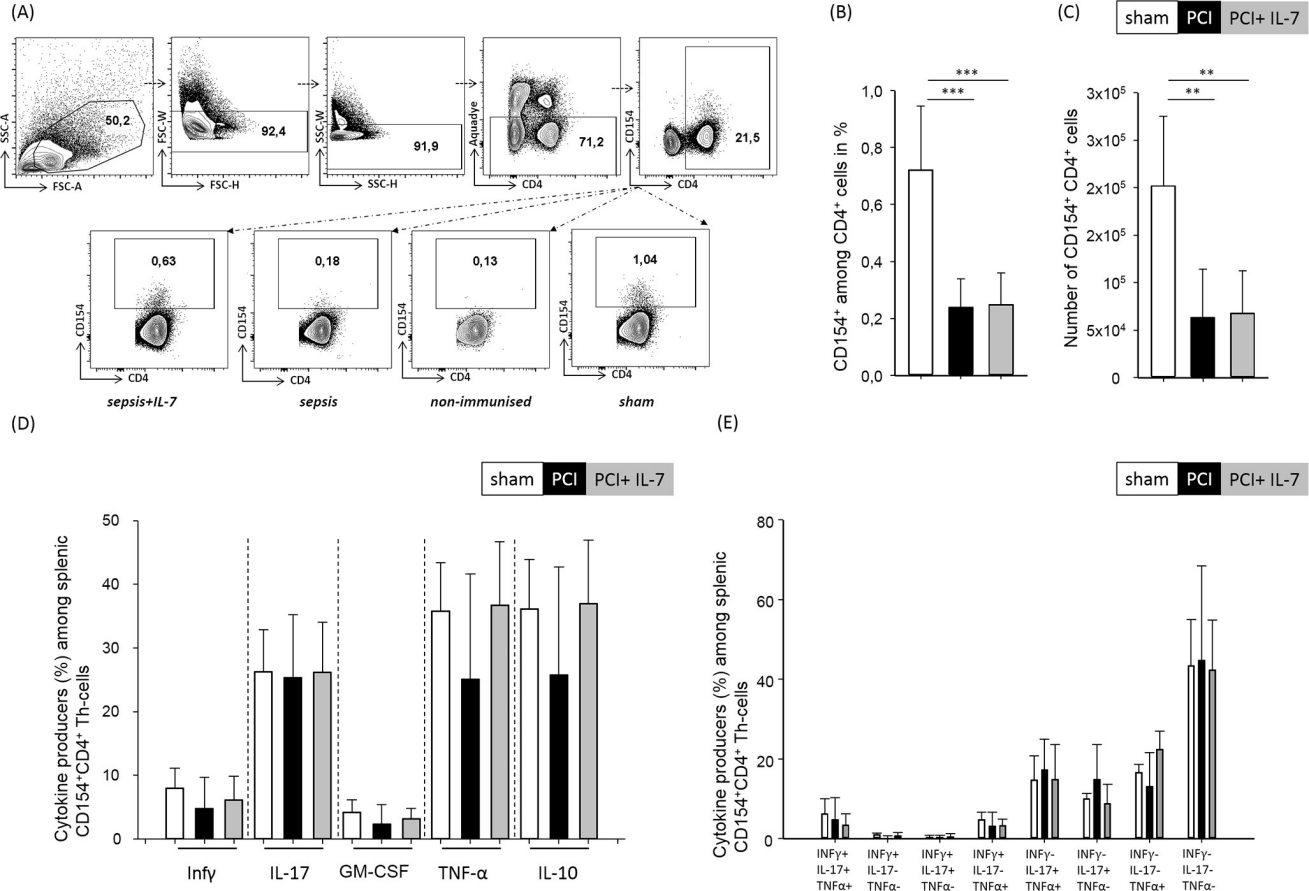
Impaired Th-cell response to fungal antigen after sepsis. Mice were injected with PBS i.p. (sham) or subjected to sepsis induction (PCI) or sepsis-induction plus IL-7 treatment (PCI + IL-7). 30 days after sepsis induction at least 5 mice from each group were immunized subcutaneously with the *Aspergillus* protein antigen GliT in complete Freund’s adjuvant. Seven days later the mice were sacrificed and the spleens were harvested. **(A)** Upon brief ex vivo culture with the antigen, GliT-specific Th cells were identified by flowcytometry using T cell-characteristic FSC/SSC criteria, doublet exclusion, exclusion of dead cells via fixable dead cell dye, and by simultaneous expression of CD4 and CD154. **(B)** Percentages of CD154^+^ among CD4^+^ cells and **(C)** total number of CD154^+^CD4^+^ cells. **(D)** Frequencies of cytokine-producing GliT-specific Th cells among CD154^+^CD4^+^ cells. **(E)** Frequencies of polyfunctional GliT-specific Th cells were analyzed by boolean gating on TNF-, IFNγ - and IL-17-producing cells.

Secondary infections, including fungal infections, contribute significantly to sepsis mortality. Efficient T-cell responses against pathogens do not only rely on a broad TCR repertoire but also on the swift induction of the appropriate effector functions. We, therefore, analyzed the T cell response against the *Aspergillus* antigen gliotoxin sulfhydryl oxidase (GliT) [33]. Thirty days after sepsis induction mice were immunized with GliT in CFA s.c.. Seven days later, the mice were sacrificed and the T cell response to GLiT was assayed by determination of CD154 expression after a brief (6 hr) period of in vitro restimulation with GliT (**Fig 5A**). Compared with sham mice the percentage and numbers of CD154^+^ GliT-specific CD4^+^ T helper cells was significantly reduced in the spleen of septic mice regardless of whether they had received IL-7 treatment or not (**Fig 5B and 5C**). Thus, the number of GliT-specific Th cells was significantly blunted in mice that had survived sepsis despite the fact that these mice had no obvious reduction in TCR-repertoire diversity (**Fig 4**). We next examined the cytokine production of GliT-specific Th cells. The frequencies of IFN-γ-, IL-17-, GM-CSF-, TNF-α, or IL-10-producing cells among the CD4^+^CD154^+^Th cells was similar in all three groups of mice (**Fig 5C**). Moreover, the frequencies of CD4^+^CD154^+^ GliT-specific Th cells that produced more than one cytokine in response to GliT was similar in all three groups of mice (**Fig 5D**). Therefore, whereas the number of GliT-specific Th cells was significantly reduced in mice that had survived sepsis, the cytokine-producing capacity within this diminished pool of cells remained intact.

## Discussion

Massive apoptosis of lymphocytes is a hallmark of sepsis. We performed a long-term examination of the quantitative and qualitative T-lymphocyte recovery after sepsis. In accordance with earlier studies [4,9,10] we found rapid, complete and stable recovery of T cell numbers after sepsis. Similar to what has been observed in recent clinical trials [28] administration of recombinant human IL-7 accelerated and enhanced the recovery of T lymphocytes. Although CD4^+^ and CD8^+^ T cells express the IL-7R at similar levels [24], we found that the beneficial effect of IL-7 treatment on T cell recovery was more pronounced for CD8^+^ T cells than for CD4^+^ T cells, which confirms and extends earlier reports on other sepsis models [25,26,44]. Recovery of T cell numbers could occur via two different, not mutually exclusive, mechanisms. The apoptotic loss of lymphocytes could be overcome by homeostatic proliferation of the surviving clones, which depends largely on IL-7 and IL-15 together with low-affinity interactions of the TCR with p/MHC complexes [39,45]. Homeostatic proliferation is thought to be the main mechanism for T cell replenishment in adult humans [39,40]. Homeostatic proliferation of surviving clones would fail to generate new TCR specificities and result in a narrowed TCR repertoire [39,45]. Moreover, data from mouse models suggest that homeostatic proliferation favours T cells with TCRs displaying higher affinity for self-peptide/MHC complexes [46]. Therefore, over the course of many years, homeostatic proliferation could result in an increased risk for autoimmune responses. Alternatively, thymic output could be increased to replenish the peripheral T cell pool with newly generated naive T cells. To date, very little is known about the relative contributions of homeostatic proliferation and increased thymic output to T cell recovery after sepsis. To estimate thymic output after sepsis we used RAG2p-GFP transgenic mice [30] in which the RAG2 promoter drives GFP expression and the GFP signal remains detectable in recent thymic emigrants for approximately three weeks after the RAG gene is no longer expressed [41]. Compatible with earlier reports on sepsis-induced thymic atrophy [47,48] we found a decreased number of GFP^+^ recent thymic emigrants (RTEs) up to 8 days after sepsis. Thereafter, the frequency of RTEs was similar in control mice, septic mice and septic mice treated with IL-7, compatible with earlier reports that had indicated that thymic output is not influenced by the number of T cells in the periphery [41]. When comparing thymectomized and euthymic mice in the CLP model, Cabrera-Perez et al. also found no influence of the thymus on quantitative T-cell recovery after sepsis [49]. IL-7 treatment did not influence the frequency of CD4^+^ or CD8^+^ of GFP^+^ RTEs, which is consistent with earlier reports on altered IL-7 signaling and responsiveness in RTEs as compared with naive T cells undergoing homeostatic proliferation [24,41,50]. In the absence of increased thymic output the recovery of T cells after sepsis-induced lymphopenia seems to occur largely via homeostatic proliferation, which might result in a diminished diversity of the TCR repertoire [39,45]. Indeed, a decreased TCR repertoire diversity was reported in sepsis patients [51]. We used next generation sequencing to determine TCR diversity in post-septic mice. Despite the fact that there was no increased thymic output these analyses did not reveal a narrowed TCR repertoire. Sepsis-survivors are burdened with an increased risk of secondary infections [4,9,10,17–19]. Similarly, post-septic mice are more susceptible to secondary infections [26,44,49,52–57]. This could be explained by the loss of particular T-cell clones after sepsis, which might be undetectable even by the analysis of large numbers of TCR sequences [49]. Alternatively, sepsis could result in functional T-cell defects [58] [12] [56]. Thirdly the sepsis-induced expansion of immunosuppressive cell populations such as regulatory T cells, IL-10 producing B-cells and myeloid derived suppressor cells (MDSC) [16] could inhibit T cell effector functions. Finally, severe infections can result in a loss or functional impairment of antigen-presenting cells, resulting in altered T-cell activation and consequently an increased susceptibility to secondary infections [59] [60] [61] [58] [62]. Which (combination) of these mechanisms is relevant for post-septic immunosuppression is currently unknown. We asked whether sepsis-induced immunosuppression was long-lasting and aimed at determining the underlying mechanisms. Immunization of mice with the fungal antigen GliT 30 days after sepsis induction revealed a strongly reduced number of GLiT-specific Th-cells in post-septic mice, regardless of whether they had been treated with IL-7 or not. Importantly, on a per-cell basis the effector functions were not different between septic mice and control mice. The GliT specific Th cells from all three groups of mice were similarly capable of producing TNF-α, IFN- γ, IL-17, GM-CSF or IL-10. Moreover, within the GliT-specific Th cell population, the frequency of cells that were capable of producing two or three cytokines simultaneously was similar in all three groups of mice. These findings support an earlier report by Markwart et al. [63], who assayed the intrinsic functions of TCR-transgenic T cells in septic mice. Our data are in contrast to earlier reports on sepsis-induced T-cell intrinsic functional impairments [58] [12] [56]. This difference may be explained by the fact that we analyzed Th cell responses 30 days after sepsis rather than during the acute sepsis episode. Our data are best compatible with a loss of GliT-specific T cell clones, which was undetectable by NGS analyses of the TCR repertoire. It is currently thought that in a naive repertoire, each individual T cell clone occurs in about 10^2^ copies. Our data are compatible with a loss of approximately 50% of the GliT-specific Th cells after sepsis. Such a loss would not be detectable with bulk methods, including NGS. Our data would also be compatible with immune suppression by IL-10 producing B cells and MDSC which are still increased in numbers 30 days after sepsis [16].

Taken together, our studies revealed a prompt reconstitution of T-lymphocyte numbers after sepsis. Whereas sensitive global analyses using NGS did not detect holes in the post-septic T-cell repertoire, our functional analyses 30 days after sepsis revealed an impaired T-cell response against a fungal antigen.

## Conclusion

Our study demonstrates long-lasting impairments in CD4^+^ T-cell responses despite rapid recovery of T-lymphocyte populations after sepsis. Whereas numerical recovery of T lymphocytes occurred quickly after sepsis and was accelerated by IL-7 treatment, the Th cell response against a fungal antigen was impaired 1 month after sepsis, regardless of IL-7 treatment. The impaired response was due to a reduced number of antigen-specific Th cells, the remaining clones were functionally intact. Our findings show that functionally relevant losses of T cell clones may go unnoticed even by sensitive global assays such as next generation sequencing of T cell receptors. Moreover, our data indicate decreased T cell responses as long-term sequelae of sepsis and suggest that this lasting immunodeficiency contributes to the increased morbidity and mortality observed in sepsis survivors.

## Abbreviations

cDNA: complementary deoxyribonucleic acid
CDR: complementarity-determining region
d: day
GFP: green fluorescent protein
GliT: gliotoxin sulfhydryl oxidase
GM-CSF: granulocyte-macrophage colony stimulating factor
HT sequencing: High-throughput sequencing
IFN- γ: Interferon gamma
IL-7: Interleukin 7
IMGT: IMMUNOGENETICS
i.p.: intraperitonea
MDSC: myeloid-derived suppressor cells
PCI: Peritoneal contamination and infection
RTE: Recent thymic emigrants
s.c.: subcutaneous
TCR: T-cell receptor
Th: T helper
TNF-α: tumor necrosis factor alpha
Treg: Regulatory T cells
